# Correction: Adapting physics-informed neural networks to improve ODE optimization in mosquito population dynamics

**DOI:** 10.1371/journal.pone.0320342

**Published:** 2025-03-10

**Authors:** Dinh Viet Cuong, Branislava Lalić, Mina Petrić, Nguyen Thanh Binh, Mark Roantree

There are errors in the Funding statement. The correct Funding statement is as follows: This work is supported by Taighde Éireann – Research Ireland through the Insight Centre for Data Analytics (SFI/12/RC/2289\_P2) and by COST Action CA20108, supported by COST (European Cooperation in Science and Technology). This research is supported by the European Union (Grant Agreement No. 101136578). The views and opinions expressed in this publication are solely those of the author(s) and do not necessarily represent the official position of the European Union or the European Commission. Neither the European Union nor the granting authority is responsible for any use that may be made of the information contained herein. Additional support was provided by the Ministry of Science, Technological Development, and Innovation of the Republic of Serbia through two Grant Agreements with the University of Novi Sad, Faculty of Agriculture (No. 451-03-66/2024-03/200117, dated February 5, 2024). The funders have no role in study design, data collection and analysis, decision to publish, or preparation of the manuscript.
