# Supplementary material for: Adapting physics-informed neural networks to improve ODE optimization in mosquito population dynamics
Source: PLoS One. 2024 Dec 23;19(12):e0315762. doi: 10.1371/journal.pone.0315762 (PMC11666042; doi:10.1371/journal.pone.0315762)
Supplement: S1 Appendix — (PDF) [file pone.0315762.s001.pdf]

Supporting Information

Error metrics

Let $y_i, i = 1 \ldots, M$ be M reference values and $\hat{y}_i, i = 1 \ldots, M$ are the corresponding predictions. Let $\mathfrak{L}$ and $\mathfrak{U}$ be the pre-defined lower and upper bounds for the values. The error metrics used in this paper, including Root Mean Squared Error (RMSE), Mean Absolute Error (MAE), Median Absolute Percenrage Error (MDAPE) and Root Mean Squared normalized Error (nRMSE) are defined as followed

$$RMSE = \sqrt{\frac{1}{M} \sum_i^M (y_i - \hat{y}_i)^2} \tag{1}$$

$$MAE = \frac{1}{M} \sum_i^M |y_i - \hat{y}_i| \tag{2}$$

$$MDAPE = \text{median}_i \left(\frac{|y_i - \hat{y}_i|}{|y_i|} \right) \tag{3}$$

$$nRMSE = \frac{RMSE}{\mathfrak{U} - \mathfrak{L}} \tag{4}$$
