# Supplementary material for: Adapting physics-informed neural networks to improve ODE optimization in mosquito population dynamics
Source: PLoS One. 2024 Dec 23;19(12):e0315762. doi: 10.1371/journal.pone.0315762 (PMC11666042; doi:10.1371/journal.pone.0315762)
Supplement: S1 Table — (PDF) [file pone.0315762.s002.pdf]

Supporting Information

ODE Parameters

Parameter	Description	Value	Unit
τ	Temperature		$^\circ C$
γ_{Aem}	Development rate of emerging adults	1.143	$days^{-1}$
γ_{Ab}	Development rate of bloodseeking adults	0.885	$days^{-1}$
γ_{Ao}	Ovipositing adult development rate	2	$days^{-1}$
$f_E(>0)$	Egg development rate	$0.16 \cdot \left(e^{[0.105(\tau-10)]} - e^{\left[0.105(38-10)-\frac{1}{5.007}(38-\tau)\right]}\right)$	$days^{-1}$
f_P	Pupa development rate	$0.021 \cdot \left(e^{[0.162(\tau-10)]} - e^{\left[0.162(38-10)-\frac{1}{5.007}(38-\tau)\right])}\right)$	$days^{-1}$
f_L	Larva development rate	$f_P/1.65$	$days^{-1}$
$f_{Ag}(>0)$	Development rate of gestating adults	$\frac{\tau-9.8}{64.4}$	$days^{-1}$
m_E	Egg mortality rate	$m_E = \mu_E$	$days^{-1}$
m_L	Larval mortality rate	$\exp[-\tau/2] + \mu_L$	$days^{-1}$
m_P	Pupa mortality rate	$\exp[-\tau/2] + \mu_P$	$days^{-1}$
$m_A(>\mu_A)$	Mortality rate of Ab,	$-0.005941 + 0.002965 \cdot \tau$	$days^{-1}$
μ_E	Minimum egg mortality rate	0	$days^{-1}$
μ_L	Minimum larval mortality rate	0.0304	$days^{-1}$
μ_P	Minimum pupa mortality rate	0.0146	$days^{-1}$
μ_{em}	Mortality rate during emergence	0.1	$days^{-1}$
μ_r	Mortality rate during bloodseeking	0.08	$days^{-1}$
μ_A	Minimum adult mortality rate	$\frac{1}{43}$	$days^{-1}$
κ_L	Carrying capacity for larvae	$8 \cdot 10^8$	$days^{-1}$
κ_P	Carrying capacity for pupae	10^7	$days^{-1}$
σ	Sex ratio at emergence	0.5	-
β	Number of eggs per Ao	$\beta_1 = 141(np), \beta_2 = 80(p)$	-
