# Supplementary figures and images for: Adapting physics-informed neural networks to improve ODE optimization in mosquito population dynamics
Source: PLoS One. 2024 Dec 23;19(12):e0315762. doi: 10.1371/journal.pone.0315762 (PMC11666042; doi:10.1371/journal.pone.0315762)

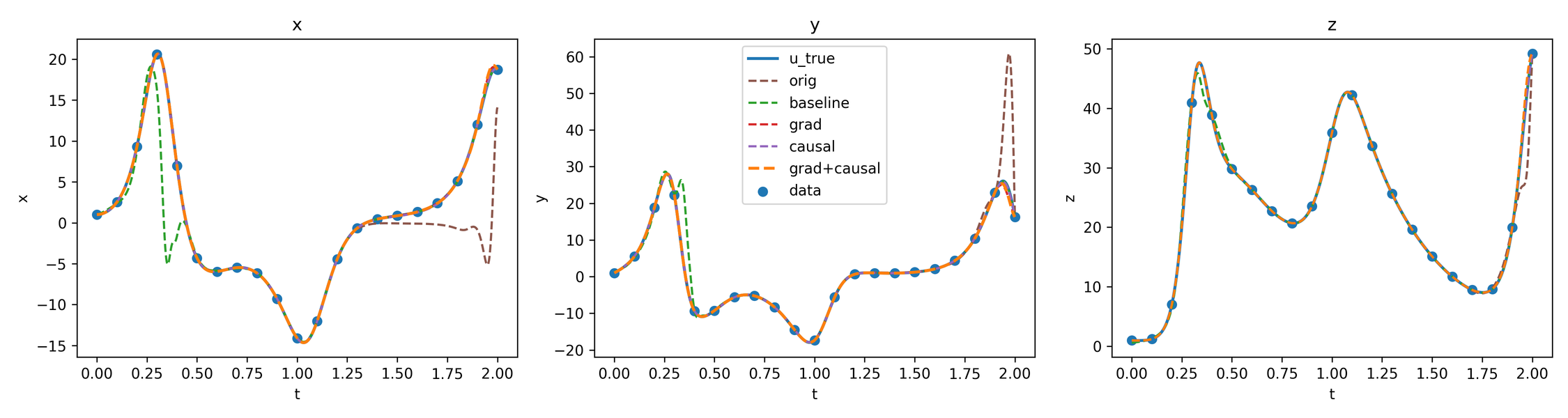

Supplement: S1 Fig — (TIF) [file pone.0315762.s003.tif]

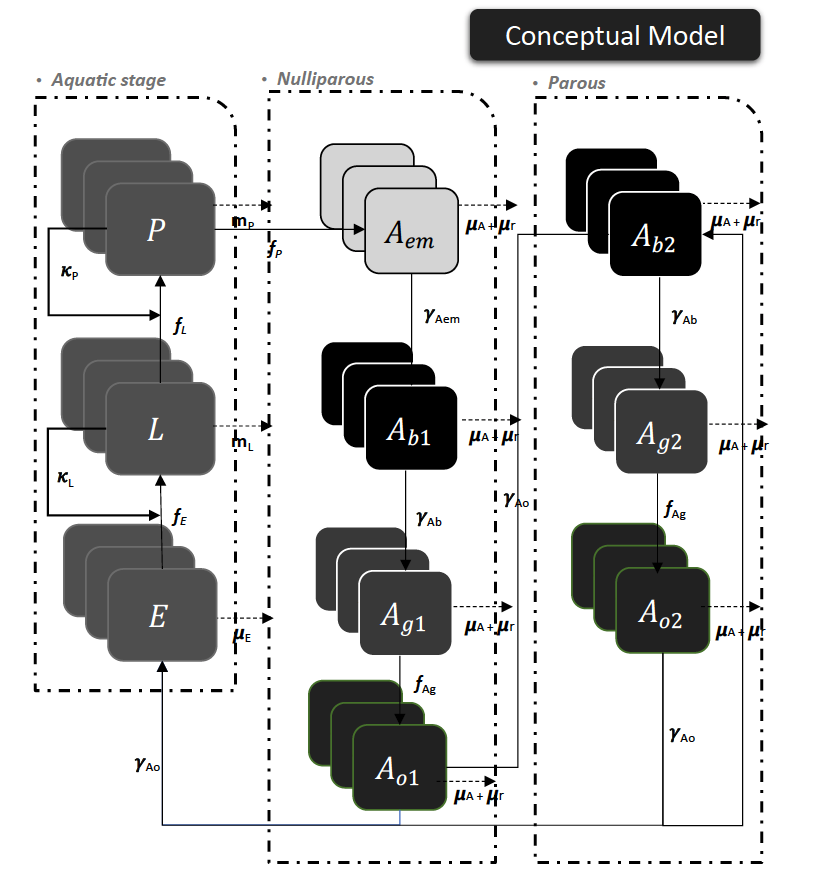

Supplement: S2 Fig — Egg (E), Larva (L), Pupa (P), Emerging Adults (Aem), Nulliparous Bloodseeking Adults (Ab1), Nulliparous Gestating Adults (Ag1), Nulliparous Ovipositing Adults (Ao1), Parous Bloodseeking Adults (Ab2), Parous Gestating Adults (Ag2) and Parous Ovipositing Adults (Ao2) (source: [11]). (TIF) [file pone.0315762.s004.tif]

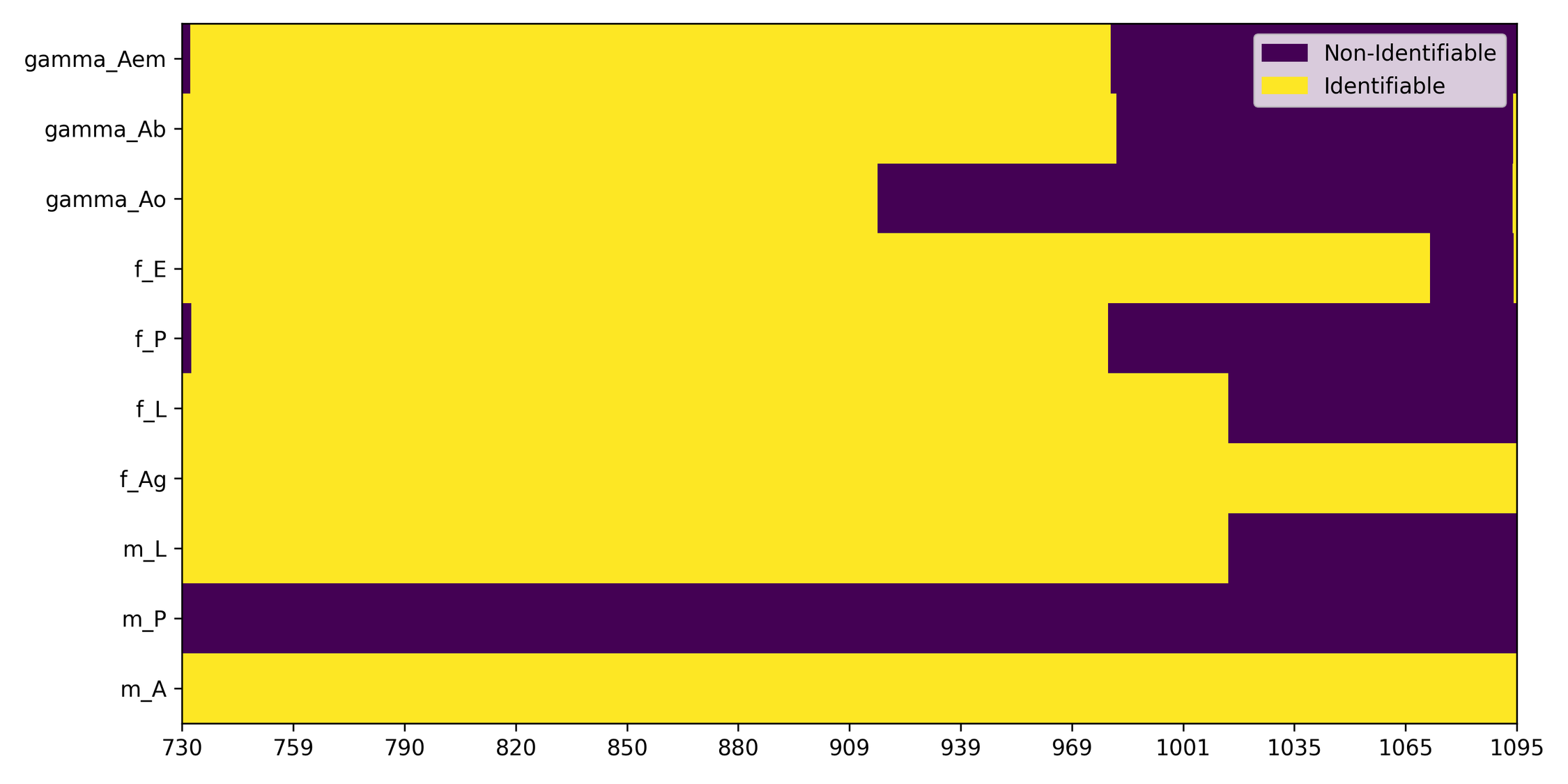

Supplement: S3 Fig — This is achieved by expressing the system as a system of linear equations, the parameters as unknown variables, and analyzing the Reduced row-echelon form of the coefficient matrix, performed separately for each time t. In the figure, identifiable parameters are defined as free parameters which can get arbitrary values. The values are rounded to 6-digit precision, aligning with the PINN’s level of precision after training. The figure explains the PINN’s inaccuracies shown in Fig 8. (TIF) [file pone.0315762.s005.tif]
